# Attentional Control as a Predictor of Response to Psychological Treatment for Depression and Relapse up to 1 year After Treatment: A Pilot Cohort Study

**DOI:** 10.1017/S1352465818000590

**Published:** 2018-10-24

**Authors:** J.E.J. Buckman, R. Saunders, P. Fearon, J. Leibowitz, S. Pilling

**Affiliations:** 1 Research Department of Clinical, Educational and Health Psychology, University College London, Gower Street, London WC1E 6BT; 1a iCope – Camden and Islington Psychological Therapies Services, Camden and Islington NHS Foundation Trust, St Pancras Hospital, 4 St Pancras Way, London NW1 0PE; 2 Research Department of Clinical, Educational and Health Psychology, University College London, Gower Street, London WC1E 6BT; 3 Research Department of Clinical, Educational and Health Psychology, University College London, Gower Street, London WC1E 6BT; 4 iCope – Camden and Islington Psychological Therapies Services, Camden and Islington NHS Foundation Trust, St Pancras Hospital, 4 St Pancras Way, London NW1 0PE; 5 Research Department of Clinical, Educational and Health Psychology, University College London, Gower Street, London WC1E 6BT; 5a iCope – Camden and Islington Psychological Therapies Services, Camden and Islington NHS Foundation Trust, St Pancras Hospital, 4 St Pancras Way, London NW1 0PE

**Keywords:** depression, relapse, predictors, treatment outcome

## Abstract

**Background:** Identifying depressed patients unlikely to reach remission and those likely to relapse after reaching remission is of great importance, but there are few pre-treatment factors that can help clinicians predict prognosis and together these explain relatively little variance in treatment outcomes. Attentional control has shown promise in studies to date, but has not been investigated prospectively in routine clinical settings with depressed patients. **Aims:** This study aimed to pilot the use of a brief self-report measure of attentional control in routine care and investigate the associations between attentional control, psychological treatment response and relapse to depression up to 1 year post-treatment. **Method**: Depressed patients were recruited from two primary care psychological treatment (IAPT) services and completed the Attentional Control Scale (ACS) alongside routine symptom measures at every therapy session. Participants were tracked and followed up for 1 year post-treatment. **Results**: Baseline ACS scores were associated with remission and residual depressive symptoms post-treatment, and relapse within 12 months of ending treatment, all independent of pre-treatment depressive symptom severity, and the latter also independent of residual symptoms. **Conclusion**: A self-report measure of attentional control can potentially be used to predict levels of depressive symptoms post-treatment and can contribute to predicting risk of relapse to depression in IAPT services, without affecting rates of therapy completion/drop-out or data completion of standard IAPT measures. However, this pilot study had a small overall sample size and a very small number of observed relapses, so replication in a larger study is needed before firm conclusions can be made.

## Introduction

Up to 80% of people with depression suffer relapses or recurrences after receiving treatment (Mueller et al., 1999). Less than half of depressed patients treated in Improving Access to Psychological Therapies (IAPT) services in England recover (NHS Digital, 2016), and in the only study to date to assess longer term outcomes in IAPT, more than half of those that reached remission experienced a relapse or recurrence within one year (Ali et al., 2017). Predictive models that attempt to identify patients that will not recover at the end of their IAPT treatment based on their pre-treatment characteristics have been developed, but despite their stability across training and hold-out test samples they have relatively weak predictive strength (e.g. Delgadillo et al., 2016; Saunders et al., 2016). Studies have also used data routinely collected during IAPT treatment to more accurately predict end of treatment symptoms and functioning (e.g. R. Saunders, J.E.J. Buckman, J. Cape, P. Fearon, J. Leibowitz and S. Pilling, unpublished observations). Work to identify those at greatest risk of relapse after IAPT treatment has also begun, with one such study showing that residual symptoms post-treatment were the strongest predictor of later relapse, but that pre-treatment characteristics were not predictive (Ali et al., 2017). A recent systematic review highlighted a number of prognostic indicators that could be assessed pre-treatment in addition to the routine measures collected in IAPT services that may help elucidate treatment and post-discharge trajectories; one such factor is attentional control (Buckman et al., 2018). The identification of prognostic indicators could further support the growing personalized medicine initiative in the treatment of depression (Chekroud and Krystal, 2015; Cohen and DeRubeis, 2018).

Attentional control involves the ability to selectively attend to information whilst ignoring distractions, quickly engage/disengage attention to/from a given stimulus, and remember and mentally manipulate information that was given a short time before (Derryberry and Reed, 2002). Attentional control has been observed to play an important role in a number of affective disorders by impacting upon information processing and attentional biases (e.g. Beck and Clark, 1997; Derryberry and Reed, 2002; Eysenck et al., 2007) and in personality disorders as it is related to the ability to exert top-down control in response to distressing or distracting external stimuli, thus affecting emotion regulation (e.g. Silbersweig et al., 2007). Several therapies have been developed to target attentional control and have proven efficacious in treating anxiety disorders; these include attention training (e.g. Wells et al., 1997) and attentional bias modification treatment (e.g., see MacLeod and Bridle, 2009); however, attentional control has been less well studied in depressed populations. There have been promising preliminary findings from one research group that reported improved clinical outcomes when using interventions (based in part on attention training: Wells et al., 1997) targeting neurobiological pathways linked to attentional control as an adjunct to usual psychological and pharmacological therapies for severely depressed patients (Siegle et al., 2007). As well as being related to treatment outcomes, attentional control has been found to remain impaired after recovery from depressive episodes (Sears et al., 2011), so it may be a potential risk factor for relapse or recurrence too. However, it may be that impaired attentional control is related to the presence of residual symptoms and that this in turn leads to an association with the risk of relapse or recurrence (Buckman et al., 2018). To date, there have been no studies known to these authors of the relationship between depressive symptoms and attentional control in routine clinical settings, and no prospective studies investigating attentional control alongside both treatment outcomes and relapse or recurrence of depression. The question, then, is whether or not levels of attentional control pre-treatment are associated with treatment response, the presence or absence of residual symptoms, and the likelihood of relapse or recurrence of depression. This pilot study aimed to assess whether using a brief self-report measure of attentional control in IAPT services could feasibly be used to help answer this question.

## Method

### Design

This was a pilot prospective cohort study of patients receiving psychological treatment for depression in two IAPT services.

### Setting and treatments

The study was conducted within two central London IAPT services, primary care mental health teams that operate as part of the UK National Health Service. The nature and purpose of IAPT services has been described in depth by Clark (2018). In brief, the services offer evidence-based psychological therapies at two levels of intensity following clinical guidelines (e.g. NICE, 2010). For depression, those with mild-to-moderately severe presentations might be offered low-intensity therapies with psychological wellbeing practitioners (PWPs). The low-intensity treatments are: guided self-help programmes facilitated by PWPs and are based on CBT and behavioural activation; group-based programmes covering similar content; or computerized CBT programmes also covering similar content, with support from PWPs. Other therapy options are offered but are much less common (NHS Digital, 2016). At any point during low-intensity therapy or for those with more severe presentations pre-treatment, it can be decided that a high-intensity therapy would be more suitable; these typically involve one-to-one 50–60 min long sessions with a qualified CBT therapist, Clinical or Counselling Psychologist. For depression a number of treatment options are available but the vast majority of patients engage in CBT. Nationally most services offer between eight and nine sessions on average across low and high intensity (Clark, 2018). The treatment outcomes in IAPT are judged and benchmarked nationally; in these calculations they include anyone that has had at least two treatment sessions irrespective of what treatment or why treatment ended. So, in this study, a minimum of two sessions is used to determine whether or not a patient received treatment.

### Participants

Eight PWPs working within the services sought consent from patients starting low-intensity treatment for depression between September 2013 and April 2014.

*Inclusion criteria.* a clinical diagnosis of depression and being in ‘caseness’ on the Patient Health Questionnaire 9-item version (PHQ-9) (a score of 10 or more) pre-treatment (Kroenke et al., 2001), and the ability to read and write in English.

*Exclusion criteria.* a score below 10 on the PHQ-9 pre-treatment; not able to give consent for the study; or not able to read and write in English.

### Measures

All IAPT services require patients to complete a range of routine measures at every appointment, (see IAPT, 2011, for details), including the PHQ-9 for depressive symptoms, the Generalized Anxiety Disorder scale (GAD-7; Spitzer et al., 2006) for symptoms of generalized anxiety, and the Work and Social Adjustment Scale (WSAS; Mundt et al., 2002) as a measure of functioning. Participants were also asked to complete the Attentional Control Scale (ACS; Derryberry and Reed, 2002) at every treatment appointment and again at follow-up. The ACS is a 20-item self-report measure, including items on focusing attention, shifting attention, and flexible control of thinking. Higher scores indicate better attentional control. The ACS has acceptable convergent and discriminant validity with pen-and-paper attention tasks, computerized tests of attentional control and personality and arousal tests, with significant correlations between the ACS and Pavlovian temperament traits, extraversion and neuroticism, energetic arousal and tension arousal (*p* < .01 in all cases), but not with recognition of emotional and non-emotional signals (Fajkowska and Derryberry, 2010). The ACS has good internal consistency (Cronbach's alpha =0.88), and moderate test–retest reliability, *r* = 0.61 over 1 month (Fajkowska and Derryberry, 2010). Although attentional control is commonly assessed using computerized tasks, the ACS was considered the best available means of measuring it in routine clinical care.

### Procedure

PWPs recruited participants from their caseloads. Participants completed all measures at every session until they ended treatment; they were tracked for one year through their electronic patient records and the study team (not the treating clinicians) then contacted them at approximately 6 and 12 months post-treatment to complete the four measures again over the telephone. If they returned to the services in the interim for a new episode of care, PHQ-9 and GAD-7 scores were recorded on their return.

### Analysis

#### Primary outcomes

(1) Feasibility of recruitment, retention and data completion of routine IAPT measures throughout the study. (2) Remission: scoring below 10 on the PHQ-9. (3) Relapse: being in remission then scoring 10 or above on the PHQ-9 at any point between 3 weeks after the end of treatment and the 1 year follow-up appointment. To be counted as a relapse the change in the PHQ-9 score had to be equal to or greater than the reliable change index score on the PHQ-9 (5 points; Löwe et al., 2004).

#### Secondary outcomes

(1) Residual depressive symptoms: post-treatment PHQ-9 scores between 5 and 9 (Kroenke et al., 2001). (2) IAPT recovery: in caseness on either the PHQ-9 or GAD-7 pre-treatment (a score of 8 or above on the GAD-7), and below caseness on both measures post-treatment (IAPT, 2011). (3) Reliable improvement then deterioration: an improvement pre-to-post treatment of at least 5 points on the PHQ-9, followed by a deterioration by at least 5 points during follow-up.

#### Primary explanatory factors

Pre-treatment ACS score was treated as the primary explanatory factor for the primary and secondary outcomes. For post-discharge outcomes the change in ACS scores (calculated by subtracting the start of treatment score from the end of treatment score) from pre-to-post treatment was considered as an alternative explanatory factor in separate models.

### Statistical methods

All analyses were performed using STATA version 13.0. (StataCorp, 2013). Univariable logistic regression models were built independently to assess the effects of potential risk factors on primary and secondary outcomes. Odds ratios (OR) and 95% confidence intervals (CI) are presented, and for continuous variables that were approximately normally distributed independent *t*-tests are also presented. Multivariable logistic regression models were built to consider potential confounding factors that were fitted into the regression models if they: were independently associated both with the pre-treatment ACS score and the outcome; could not have been potentially caused by levels of attentional control pre-treatment; resulted in a change in the OR related to pre-treatment ACS score; and if they improved the regression models based on the likelihood ratio test comparing the model with the potential confounder to the same model but without the confounder. Models were adjusted for factors assumed *a priori* to affect the relationships between ACS scores pre-treatment and the outcome, e.g. adjustment was made for pre-treatment PHQ-9 scores in models of post-treatment outcomes, and for residual symptoms in models of post-discharge outcomes. The tolerance and variation inflation factor (VIF) statistics (Garson, 2012) were considered for each model to assess the possible effects of multi-collinearity. Overly influential data points were considered by plotting standardized Pearson residuals, deviance residuals and Pregibon leverage each against the predicted probabilities by the unique participant identification number.

## Results

### Descriptive statistics

Sixty-nine patients were recruited to this pilot study. As data were pseudonymized for the study team with the IAPT staff member's details removed, it was not possible to compare those recruited by the eight PWPs taking part in the study and those on their caseloads that did not get recruited to the study. However, there were no significant differences in the baseline characteristics of those recruited and the population attending the services with all therapists over the study period (Table 1).

**Table 1.** Demographic and baseline clinical characteristics of pilot study participants and population of attendees at the IAPT services over the study period

		Pilot study *n* (%)	IAPT population *n*	*p*-
Characteristic		or mean (*SD*)	(%) or mean (*SD*)	value*
***n***		69	3307	
**Demographics**				
Age at referral (years)		34.78 (12.08)	38.03 (13.38)	.082
Sex	Male	19 (27.54)	987 (33.25)	.259
	Female	50 (72.46)	1977 (66.61)	
Ethnicity	Any White ethnicity	54 (78.26)	1713 (70.81)	.179
	Non-White	15 (21.74)	706 (29.19)	
**Pre-treatment clinical factors**				
**Self-reported measures**	PHQ-9 score	16.84 (4.54)	17.57 (4.60)	.740
	GAD-7 score	13.42 (4.46)	14.72 (4.37)	.061
	WSAS score	21.76 (7.87)	20.67 (9.29)	.334
	ACS score	45.28 (8.58)	n/a	
Prescribed anti-depressants	Yes	31 (53.45)	1319 (43.72)	.139
	No	27 (46.55)	1698 (56.28)	

*From chi-squared tests for count variables and from independent samples *t*-tests for comparisons of means.

*Treatment:* Study participants attended between two and 18 treatment sessions [median (IQR) = 5.00 (4–6)], all started treatment at low intensity, with the majority receiving guided self-help (41: 59.42%), then group-based therapy (22: 31.88%), computerized CBT (6: 8.70%) and other therapy (1: 1.45%). Eight participants (11.59%) were stepped up to high-intensity during treatment. The majority of participants (53.45%) were also prescribed psychotropic medications during their treatment in the IAPT services.

**Figure 1.**
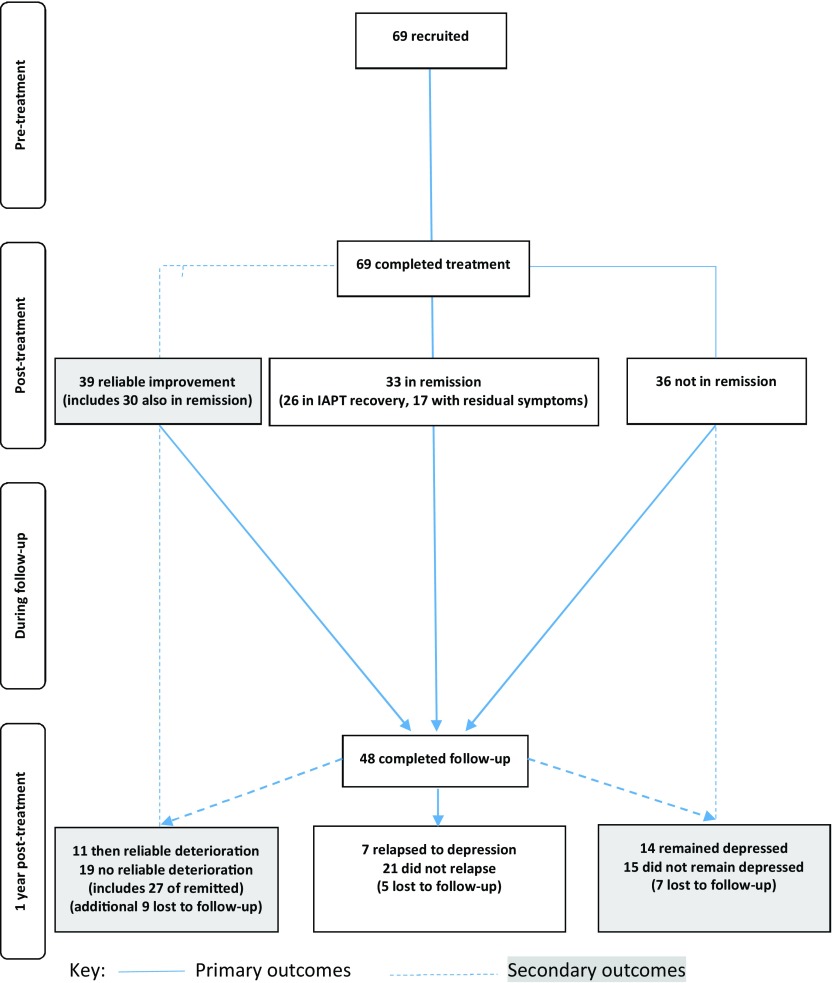
Participant flow throughout study.

*Post-treatment:* Thirty-three participants (47.83%) reached remission, 51.15% of these had residual symptoms. Twenty-six participants (40.63%) met IAPT recovery criteria; see Fig. 1.

*Follow-up:* Forty-eight participants (69.56%) completed a 1-year post-treatment follow-up appointment. Seven participants returned to the services for further treatment, six received psychological therapy elsewhere, and 16 were taking anti-depressant medications (ADM), of which five began ADM during follow-up. Seven of the 33 participant that reached remission post-treatment (21.21%) relapsed to depression, and a further four participants returned to caseness but did not meet full criteria for relapse (Table 2). Eleven of the 39 participants that had reliably improved at the end of treatment (36.67%) suffered a reliable deterioration on the PHQ-9 over follow-up, and 14 of the 36 participants that did not reach remission post-treatment (38.89%) remained depressed throughout follow-up; see Fig. 1.

**Table 2.** Relationship between baseline ACS score and each of the primary and secondary outcomes

	Present		Absent		
		Mean (*SD*)		Mean (*SD*)	*t*-test
Outcome	*n* (%)	ACS score	*n* (%)	ACS score	*p*-value
Remission	33 (47.83)	48.85 (6.43)	36 (52.17)	42 (9.06)	*p* < .001
Relapse	7 (25.00)	42.43 (3.46)	21 (75.00)	49.43 (4.71)	*p* = .001
Residual symptoms	18 (56.25)	46.33 (5.19)	14 (43.75)	50.79 (5.60)	*p* = .027
IAPT recovery	26 (40.63)	49.31 (6.85)	38 (59.38)	42.18 (8.50)	*p* < .001
Reliable improvement then	11 (36.67)	43.09 (4.70)	19 (63.33)	49.05 (6.22)	*p* = .010
deterioration					
Remained depressed	14 (53.85)	42 (7.86)	12 (46.15)	46.33 (7.63)	*p* = .169

### Primary and secondary outcomes

*Feasibility of data collection and follow-up.* There was a 100% retention rate during treatment and 70% retention over the 1-year follow-up period, slightly exceeding our expectations of 20% drop-out over 6 months and a further 20% between 6 and 12 months. There was a difference in the loss-to-follow-up by post-treatment status; seven of the 36 not in remission were lost to follow-up, compared with 14 that reached remission or reliably improved. One concern of the clinical teams prior to commencing the study was that the addition of the ACS at each session could lead to a drop in the rate of data completion of the routine IAPT measures. IAPT services aim to have at least 98% of participants with paired PHQ-9 and anxiety measures (usually GAD-7) pre–post treatment; in this study just one participant did not have paired pre–post measures (1.45%), suggesting adequate data completion of the routine measures despite the addition of the ACS.

*Treatment response*: Scores on the ACS and PHQ-9 were moderately correlated pre-treatment (*r* = –0.52, *p* < .001) and strongly correlated post-treatment (*r* = –0.62, *p* < .001). There was a moderate correlation between the change in PHQ-9 scores and change in ACS scores pre-to-post treatment (*r* = 0.45, *p* < 001) (strength of correlations taken from conventions by Swinscow and Campbell, 2002).

ACS scores were related to outcomes at the end of treatment: the mean (*SD*) ACS score pre-treatment was lower for those that did not achieve remission [42 (9.06)] compared with those that did [48.85 (6.43)], *p* < .001 (see Table 2), standardized mean difference = –0.87. There was also a difference in the direction and degree of change in the ACS scores; the mean (*SD*) change in ACS score for those not reaching remission was –1.875 (6.65) points (the minus number indicating a decrease in ACS score, thus a decrease in attentional control pre-to-post treatment), compared with 2.43 (6.17) points for those in remission (*p* = .011). A positive change of any degree in ACS score pre-to-post treatment was associated with a greater probability of remission [OR (95% CI) = 3.80 (1.32–10.90), *p* =.013]. For every one point increase in ACS score pre-to-post treatment after controlling for PHQ-9 scores pre-treatment, the odds of reaching remission increased [OR (95% CI) = 1.27 (1.09–1.45), *p* = .002].

Mean ACS scores pre-treatment were higher for those that achieved IAPT recovery compared with those that did not (see Table 2). Pre-treatment ACS scores were predictive of IAPT recovery univariably but the association was not significant after controlling for pre-treatment PHQ-9 scores [OR (95% CI) = 1.08 (0.99–1.18), *p* = .081]. However, the change in ACS scores pre-to-post treatment was associated with IAPT recovery after controlling for pre-treatment PHQ-9 scores [OR (95% CI) = 1.18 (1.05–1.34), *p* = .006].

*Residual symptoms*. Seventeen of the 33 participants that reached remission post-treatment (51.51%) had residual symptoms, and their mean ACS score pre-treatment was lower than those that did not have residual symptoms (Table 2). All but one of those that relapsed over the study period had residual symptoms post-treatment.

*Outcomes over follow-up*. The mean pre-treatment ACS score in those that went on to relapse [42.43 (3.46)] was lower than the mean in those that did not relapse [49.43 (4.71)] (Table 2); standardized mean difference = –1.57. Pre-treatment ACS scores were associated with relapse and with reliably improving pre–post treatment then reliably deteriorating post-treatment to follow-up, after controlling for both baseline and end of treatment PHQ-9 scores (or residual symptoms), relapse [OR (95% CI) = 0.24 (0.06–0.96), *p* = .043]; reliable improvement then reliable deterioration [OR (95% CI) = 0.72 (0.54–0.97), *p* = .030]. There were no other variables measured at baseline that were significantly associated with both the outcome over follow-up and pre-treatment ACS score, and none that significantly improved the logistic regression models.

The follow-up outcomes among the 36 participants that did not reach remission post-treatment were not significantly associated with baseline ACS scores (*p* = .710), or changes in ACS score pre-to-post treatment (*p* = .310). Mean pre-treatment ACS scores were not significantly different between those that did and did not go on to remain depressed over the course of the study.

Inspection of plots of standardized residuals and deviance suggested no overly influential data points so no participants were removed from the multivariable models. There was no apparent problematic multi-collinearity, with tolerance and VIF values acceptable for all models (0.60–1.07 and 1.60–2.24, respectively).

## Discussion

### Key findings

This study found that a representative sample of participants could be recruited and retained in a cohort for up to one year after completing treatment, and that levels of attention control could be measured with the ACS without impacting on the rates of completion of the routine symptom measures in the IAPT services. In addition, pre-treatment ACS scores changed pre- to post-treatment; they improved for those that reached remission and did not improve or got worse for those that failed to reach remission. The pre-treatment scores were associated both with response to IAPT psychological treatment for depression and outcomes during follow-up, including relapse to depression up to one year post-treatment, independent of pre-treatment depressive symptom severity and post-treatment residual symptoms.

### Interpretation

The results of this small pilot study suggest that it is feasible to measure attentional control with a self-report measure as part of routine care in IAPT services, and trace and follow up those that complete the measure for up to a year post-treatment. However, more work may need to be done to ensure that those in remission or reliably improved post-treatment are not lost to follow-up and minimize related selection biases. The association between baseline levels of attentional control and depressive symptoms, and changes in both attentional control and depressive symptoms throughout therapy offers support to evidence suggesting that attentional control plays an important role in the onset and maintenance of affective disorders (e.g. Derryberry and Reed, 2002; Eysenck et al., 2007). Finding that changes in the levels of self-reported attentional control for those that experienced remission were considerably higher than for those that did not reach remission lends support to the nascent literature on the effectiveness of treatments able to impact upon functioning in this area (e.g. Hakamata et al., 2010; Siegle et al., 2007). Perhaps of greater interest to IAPT services, finding that baseline levels of attentional control were associated with psychological treatment for depression outcomes and could potentially be associated with relapse even after controlling for depressive symptoms, suggests that there is potential for the use of ACS in IAPT services to help improve predictions of treatment and post-discharge trajectories. This is something that has not been reported in the literature before particularly as it is a potential pre-treatment risk factor for relapse, but is broadly in keeping with theories about attentional control and depression (Buckman et al., 2018; Sears et al., 2011). It remains to be seen whether these findings would be replicated in a larger sample and indeed whether adding pre-treatment ACS scores to the predictive models built on IAPT populations already (e.g. Delgadillo et al., 2016; Saunders et al., 2016) would in fact improve predictions, but it seems that this is possible and worth investigating further. To elaborate: findings from this very small sample should not be interpreted as indicating that pre-treatment attentional control is associated with relapse independently of pre-treatment depressive severity and residual symptoms. Instead, they are suggestive of an association that is worth exploring further in a larger study, and give an indication of the maximum likely effect size and therefore the minimum sample size required to investigate this thoroughly. If the preliminary findings from this pilot study were replicated in a larger sample it is plausible that measures of attentional control might be used in routine practice to allow clinicians to identify patients at greater risk of not reaching remission or of relapse during their acute-phase therapy, and it might be that clinicians could use their knowledge of a patient's level of attentional control to tailor their interventions targeting attentional control in therapy to potentially reduce the risk of these outcomes. Furthermore, as the ACS is a brief self-report measure it could potentially be used regularly throughout therapy to determine whether or not interventions are indeed affecting levels of attentional control and further inform the course of therapy.

### Strengths and limitations

This is the first study to have prospectively measured attentional control and symptoms of depression in the context of a routine clinical practice. In so doing the study has shown that attentional control can be measured quickly with a self-report measure, with potential utility in predicting psychological treatment response and very tentatively (given the number of relapses in the cohort), there could potentially be utility in using the ACS to predict relapse post-treatment. In addition, unlike many previous studies of predictors of treatment response and relapse to depression, the present study focused on routine psychological therapy service patients rather than those seen in specialist settings, many of whom had not only unipolar depression but also comorbid anxiety problems. As a result, the findings if replicated in a larger study could be generalizable to the wider population presenting with depression in routine care settings.

It is possible that significant selection biases may have been introduced as this pilot did not randomly select participants and the convenience sample of 69 participants represents only a small number of the overall population treated for depression in the services over the study period. However, no differences were found on any of the demographics or pre-treatment symptom measures between the study sample and the population from which the sample was drawn. Furthermore, this pilot study attempted to consider the potential use of a low-cost and brief screening measure of attentional control to be used to better inform considerations of post-treatment outcomes and relapse in a routine care setting, so it was beyond the scope of this study to randomly select or randomly assign patients to treatment. A study that randomly assigns those with differing levels of attentional control to treatments specifically targeting or not targeting attentional control may help to answer questions of the utility of knowing about a patient's level of attentional control pre-treatment; they might allow for prescriptive considerations rather than the prognostic indication we have considered in this study.

Confounding by a number of potential factors cannot be ruled out as a potential explanation for our findings. While we controlled for a number of potential confounders in multivariate regression analyses it is likely that further unmeasured confounding factors impacted on our findings, e.g. we had no measure of intelligence/IQ, no data on the number of previous depressive episodes or the number of previous IAPT treatments in which remission was/was not achieved. All have previously been related to depression treatment outcomes (e.g. Cohen and DeRubeis, 2018; DeRubeis et al., 2014) or relapse (Buckman et al., 2018) and may feasibly be independently related to attentional control.

The biggest limitation of this pilot study is the small number of participants that experienced a relapse. As a result, any conclusions regarding relapse must be treated with caution and only replication of these findings in a larger sample will be sufficient to draw sound conclusions about the ability of services to use the ACS at baseline assessment as a tool to help predict who is most likely to achieve remission and who might relapse to depression.

We did not have a continuous measure of depressive symptoms over follow-up (such as the Longitudinal Interval Follow-up Evaluation – LIFE; Keller et al., 1987) and therefore may have missed some relapses; using such a measure would allow for more accurate data on remission and recovery so could then be used to capture both relapses and recurrences over follow-up. Without this continuous measure it is possible that some of those classified as having had a relapse here may have had a recurrence.

### Conclusions and implications

This pilot study has shown that it is feasible to recruit IAPT patients to a cohort study and use the ACS to measure attentional control as a potential predictor of treatment outcomes and outcomes up to one year post-treatment. Further, it has shown that it was possible to do so without a loss of data completion of the routine symptom measures used in IAPT. This was the first prospective study in a routine clinical setting to show that levels of attentional control change in accordance with levels of depressive symptoms, and that those pre-treatment levels could be associated with outcomes independently from depressive symptoms. However, as this was only a pilot study with a small number of participants and a very small number of relapses, replication in a larger study either randomly selecting participants or using the ACS with the whole population of patients attending services for treatments for depression is needed before sound conclusions can be drawn. In addition, trials of treatments offered to those identified as having an increased risk of not reaching remission or of relapse are needed before any conclusions can be drawn on the utility of using the ACS to identify these outcomes. If findings were to be replicated in larger studies it is feasible that the ACS could be used to inform treatments by using interventions considered to help improve attentional control, and by being measured regularly throughout treatment there might be an opportunity for regular feedback and adjustment of therapy to ensure attentional control is in fact improving; as such it may be possible to increase the odds of remission and reduce the probability of relapse to depression.
